# Systemic Inflammatory Cytokines Predict the Infectious Complications but Not Prolonged Postoperative Ileus after Colorectal Surgery

**DOI:** 10.1155/2018/7141342

**Published:** 2018-03-06

**Authors:** G. S. A. Boersema, Z. Wu, A. G. Menon, G. J. Kleinrensink, J. Jeekel, J. F. Lange

**Affiliations:** ^1^Department of Surgery, Erasmus University Medical Center, Rotterdam, Netherlands; ^2^Ward I of Gastrointestinal Cancer Center, Key Laboratory of Carcinogenesis and Translational Research, Ministry of Education, Peking University Cancer Hospital & Institute, Beijing 100142, China; ^3^Department of Surgery, Havenziekenhuis, Rotterdam, Netherlands; ^4^Department of Neuroscience, Erasmus University Medical Center, Rotterdam, Netherlands

## Abstract

**Aim:**

Postoperative ileus (POI) is common after surgery. Animal studies indicate that the POI mechanism involves an inflammatory response, which is also activated during postoperative complications. This study aimed to determine whether inflammatory biomarkers might facilitate an early detection of prolonged POI (PPOI) or infectious complications.

**Methods:**

Forty-seven adult patients who underwent oncological colorectal surgery were included. They filled out a perioperative diary to report their gastrointestinal symptoms. Blood samples were collected preoperatively, and on postoperative day (POD) 1 and 3. Levels of leucocytes, C-reactive protein (CRP), interleukin (IL)-6, TNF-*α*, and IL-1*β* were analyzed.

**Results:**

Patients with PPOI had significantly longer stay in hospital than patients without (13.6 ± 10.5 versus 7.4 ± 3.2 days, *p* < 0.001); they also had higher levels of IL-6 ratios, leucocytes, and CRP levels, but did not reach significance. Higher levels of postoperative IL-6 and CRP levels (*p* < 0.05, resp.) were found in patients with infectious complications. The receiver operating characteristic (ROC) analysis found better diagnostic values of IL-6 ratio on both POD 1 and 3 than that of CRP (POD 1: ROC 0.825, *p* < 0.001).

**Conclusion:**

Blood levels of inflammatory cytokines cannot predict PPOI after colorectal surgery. Instead, postoperative IL-6 changes may predict the infectious complications with a better diagnostic value than the current leukocytes or CRP tests.

## 1. Introduction

Surgical resection is still the cornerstone of colorectal cancer treatment. Nevertheless, colorectal surgery is associated with a high morbidity rate of 24–43% [1–3], which significantly compromises a fast recovery after surgery and quality of life after discharge. Infectious complications including surgical site infection and anastomotic leakage are the major causes of postoperative morbidity and mortality [4]. Moreover, many patients also develop postoperative ileus (POI) characterized by a transient impairment of bowel function and reduced motility. In some of them, prolonged POI (PPOI) is diagnosed when POI does not resolve after 5 postoperative days or recurs after an apparent resolution. Such delayed recovery of bowel function leads to other serious outcomes such as longer hospitalization, hospital-acquired infections, pulmonary compromise, and a large increase of medical cost as well [5].

Many studies on animal models have revealed that the mechanism of POI includes an inflammatory response caused by the intestinal manipulation and surgical trauma [6–8]. Therefore, inflammatory markers such as interleukin (IL)-1*β*, IL-6, TNF-*α*, and C-reactive protein (CRP) have been suggested to be valuable for the early detection of POI. Previous studies reported that the levels of IL-1*β*, IL-6, and TNF-*α* in PPOI patients were significantly higher at postoperative day 5 in abdominal drain fluid than that in normal recoveries [9, 10]. However, due to the wide application of the ERAS (enhanced recovery after surgery) program, peritoneal drainage is no longer routine practice in colorectal patients. In such cases, measuring systematic levels of the inflammatory cytokines seems to be a promising alternative since it can be easily integrated into postoperative blood tests.

This approach is supported by animal studies, which have revealed that elevation of the inflammatory cytokines is also detectable in blood samples in addition to a localized change [6, 7]. Nevertheless, clinical data to support this are still not yet available. Moreover, it is important to note that the classic proinflammatory response is also activated in infectious complications, and increasing levels of the inflammatory cytokines were also reported in these complications [11–16]. To this end, we conducted a prospective cohort study in patients underwent colorectal surgery. In this study, we analyzed the systemic inflammatory markers in perioperative blood samples. The primary goal of this study was to investigate whether the perioperative inflammatory cytokine levels can predict PPOI. Secondarily, we also tried to associate the cytokine levels with the infectious complications.

## 2. Method

### 2.1. Study Population and Design

Adult patients admitted to the Academic Colorectal Cancer Center, Havenziekenhuis, Rotterdam, who underwent oncological colorectal surgery, were included after informed consent. In total, 50 patients were planned to be included in this prospective cohort during the period of November 2013 and November 2014. In accordance with the Dutch law on medical research in humans, this study was approved by the Medical Ethical Committee of the Erasmus University Medical Center, Rotterdam, Netherlands (permit number: MEC-2013-246, NL43053.078.13) and patients gave their written consent after receiving oral and written information.

All patients were asked to fill out a questionnaire before surgery and every day after surgery until postoperative day (POD) 7. The questions refer to their food and fluid intake, bowel movements and defecation, gastrointestinal symptoms, and visual analogue scale (VAS) pain score. Data collection included age, gender, body mass index (BMI), American Society of Anesthesiologists (ASA) score, medication use, smoking, operative procedure, and postoperative complications including anastomotic leakage, fascia dehiscence, surgical site infection (SSI), urinary tract infection, pneumonia, and postoperative course.

### 2.2. Selection of Variables and Definitions

To ensure the objectivity of the primary endpoint, PPOI was not diagnosed by the participating surgeons but via the retrospective review of the patient diary and medical record. The participating doctors diagnosed the other complications based on the criteria referred from the literature [17–19] (see Table 1).

### 2.3. Blood Sample Analysis

Peripheral blood was drawn from each patient before surgery (baseline) and on the first and third postoperative days in the morning, together with the routine blood tests. Leucocytes and CRP measurements were part of the standardized care and the outcomes were retrieved from the medical chart. Blood samples were centrifuged and plasma was stored at −80°C into two aliquots for each sample. Enzyme-linked immunosorbent assays (ELISAs) were performed according to manufacturer's instructions to quantify the concentrations of systematic inflammatory markers IL-6 and TNF-*α* (PeproTech Inc., Rocky Hill, USA) and IL-1*β* (R&D Systems, Minneapolis, MN, USA) in blood plasma.

### 2.4. ERAS Protocol

All patients were treated according to the ERAS protocol. Two hours before surgery patients preoperatively received a carbohydrate-loaded drink. In some cases of low anterior resection, an enema was given under prescription of the surgeon. In general, left-sided colectomy and (low) anterior resections received bowel preparation with 2 liters of Macrogol 3350 (Klean-Prep 69 gr, Norgine Ltd., Harefield, United Kingdom). Immediately after surgery, nasogastric tubes were removed and patients are allowed to move or drink fluid food. Normal diet was offered from the first postoperative day and so on.

### 2.5. Statistical Analysis

The statistical analysis was carried out using the Statistical Package for the Social Sciences (SPSS Inc., Chicago, USA, version 21.0 for Windows). Demographic data were presented in *n* (%), mean (SD), or median (95% confidence interval). Mann–Whitney *U* test, chi-square test, Pearson correlation test, and receiver operating characteristic (ROC) analysis were employed according to a proper indication. A 2-tailed *p* value < 0.05 was considered to indicate statistical significance.

## 3. Results

Between October 2013 and November 2014, 54 patients were included. Among them, three patients were excluded because of protocol violations of the inclusion criteria, and four patients retracted the informed consent. In total, 47 patients were included for analysis.

### 3.1. PPOI versus Non-PPOI

In total, 72% (34/47) of the patients recovered from POI within five postoperative days (POD 5) and were assigned to the non-PPOI group; 28% (13/47) patients recovered on or after 6 days postoperatively (8/13) or had recurrence of POI (5/13) and were therefore defined as PPOI. Univariate analysis showed a similar baseline and operative characteristics in the patients with or without PPOI (Table 2).

IL-6 levels were detectable and measurable in all the samples. However, TNF-*α* and IL-1*β* were not detectable in the majority of samples. The detailed proportions of detectable samples are listed in Supplementary Table S1.

The absolute median values of cytokines of positive samples are presented in Supplementary Table S2. In the detected samples, we found several samples with substantially higher levels of cytokines compared with other samples, resulting in a large variation in results. We also found that cytokine levels of these patients remained high after surgery. Therefore, a cytokine ratio was calculated with the following equation: ratio POD1 (or 3) = cytokine level on POD1 (or 3)/cytokine level before surgery for further analysis. Cytokine levels and ratios describe the ratio of cytokine levels at postoperative days 1 and 3 divided by the preoperative cytokine level.

**Figure 1:**
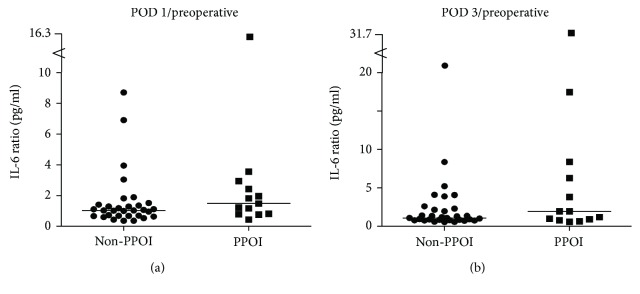
(a) and (b). IL-6 ratio in normal recoveries (non-PPOI) versus PPOI patients, every single dot represents a patient, the line indicates the median, and there are no significant differences.

The PPOI group showed higher IL-6 ratios on POD 3: 5.90 ± 9.11 than in the non-PPOI group: 2.44 ± 3.84 (Figure 1). Due to a low number of valid values, we found no differences in IL-1*β* ratio and TNF-*α* ratio between the two groups.

**Figure 2:**
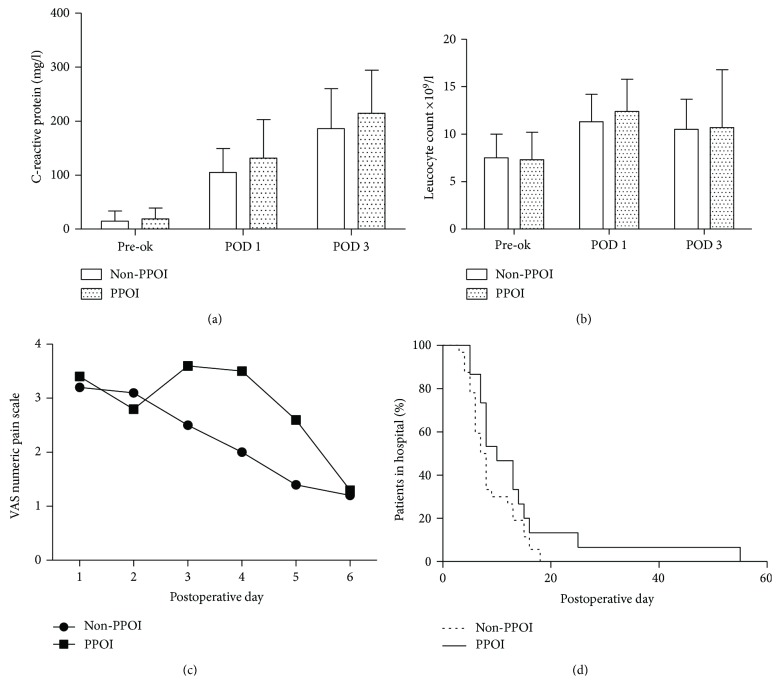
The leucocyte count and CRP and VAS scores in non-PPOI patients versus PPOI. In panels (a) and (b), bars represent the mean and error bars the SD. There are no significant differences. Panel (c) presents the VAS (visual analogue scale for pain) score, from postoperative day 1 up to postoperative day 6. Panel (d) presents patients with or without PPOI and the time in days of being ready for discharge. Patients with PPOI had a significantly longer hospital stay *p* < 0.001.

Both leucocytes and CRP were higher in the PPOI group, but there were no significantly differences between non-PPOI and PPOI groups at any time point (Figure 2). Also, a higher postoperative VAS score was seen in the PPOI group, though no statistical difference was observed.

In total, 13.0% (6/46) were diagnosed with colorectal anastomotic leakage. In the PPOI group, a significantly higher percentage of anastomotic leakage was seen, 38.5% (5/13) versus 3.0% (1/33) in the non-PPOI group, *p* = 0.005. The hospital stay duration was significant longer in PPOI patients 13.6 ± 10.5 versus 7.4 ± 3.2 in the non-PPOI cases, *p* < 0.001 (Figure 2(d)).

### 3.2. Infectious versus Non-Infectious Complication

**Figure 3:**
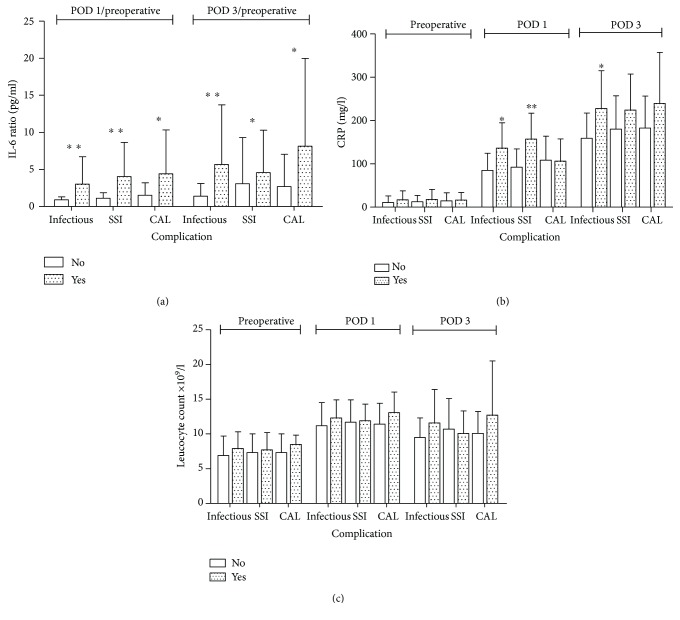
Comparison of IL-6, leucocyte count, and CRP between the patient group with infectious complication(s) (SSI, AL, pneumonia, UWI) and without infectious complication or with or without SSI (surgical site infection) or with or without CAL (colorectal anastomotic leakage). Panel (a) shows that all IL6 ratios are significant higher on both time points between all three groups; the infectious group POD 1 *p* < 0.001 and POD 3 *p* = 0.001, SSI; POD 1 *p* = 0.001 and POD 3 *p* = 0.017, CAL; POD 1 *p* = 0.027 and POD 3 *p* = 0.050. (b) On POD 1 and POD 3, the CRP levels were significantly higher in the infectious complication groups (POD 1 *p* = 0.009, POD 3 *p* = 0.008). In the SSI groups, CRP levels were significantly higher in patients with SSI compared to patients without SSI on POD 1, *p* < 0.001. Also in the groups with CAL had higher numbers of CRP though not significant. (c) Although the leucocyte count is higher in the infectious and CAL groups, there were no significant differences. Bars represent the mean, error bars, and the SD; *p* values are indicated with an asterisk; ^∗^ *p* value ≤ 0.05, ^∗∗^ *p* value ≤ 0.001.

There were no significant differences between the baseline and surgical characteristic comparison of patients with and without infectious complications (Supplementary Table S3). Different from the PPOI patients, patients with the infectious complications had significantly higher IL-6 ratios and CRP levels on POD 1 and POD 3 (*p* < 0.05, respectively, Figure 3(a)), while the leucocyte count, though also higher, was not significantly different from patients without infectious complications, Figure 3(c). Further detailed analysis showed that significantly higher levels of IL-6 ratios on POD 1 and POD 3 were found in SSI and colorectal anastomotic leakage (CAL) patients as illustrated in Figure 3(a), while the differences in CRP were not significant (Figure 3(b)). No differences were observed in IL-1*β* and TNF-*α* ratios between the groups.

**Figure 4:**
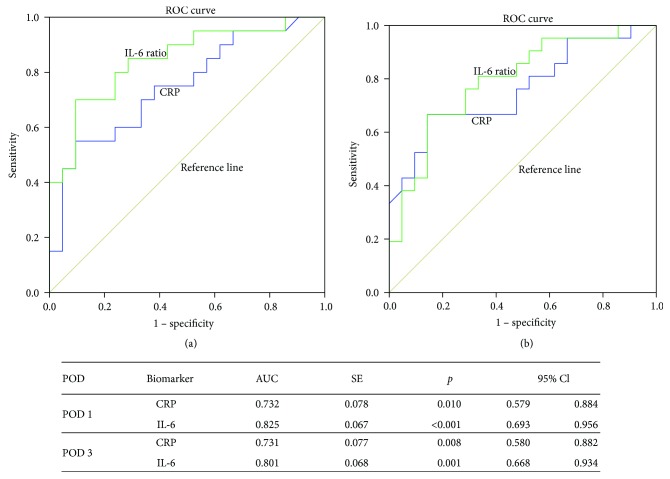
ROC analysis showed CRP and IL-6 ratio on POD 1 (a) and POD 3 (b), on both days; the AUC was higher in IL-6 ratio.

We performed the ROC analysis to determine the diagnostic value of CRP and IL-6 ratio in detection of infectious complications. Both on POD 1 and POD 3, IL-6 ratio had a larger area under curve (AUC) than that of CRP (Figure 4). Further analysis showed that the diagnostic value was achieved on POD 1 with a cutting-off point of 1.21 of IL-6 ratio, which yielded a sensitivity of 76% and a specificity of 86%. Although the sensitivity was relatively low (43%), a cutting-off point of 1.93 on POD 1 reached a specificity of 100%, meaning all patients with an IL-6 ratio higher than 1.93 on POD 1 were diagnosed with infectious complications later on.

## 4. Discussion

The importance of developing effective strategies to predict and eventually to treat the postoperative complications cannot be overemphasized [20]. In this study, we investigated the association between the inflammatory cytokines and the postoperative complications. We found that systematic changes of IL-6 predicted the infectious complications but not prolonged POI after colorectal surgery.

To develop the effective strategies, researchers depend on the translational knowledge from animal studies, which have been continuously contributing to the understanding of POI mechanism. Several experimental studies have reported the important role of IL-6 in the development of POI. Even little manipulation of the bowel induces activity of IL-6, which results in activation of nitric oxide and prostaglandins and causes migration of leucocytes into the circular muscle of the bowel and ends up with PPOI eventually [21–24]. However, with fruitful data obtained from animal studies, clinical attempts to predict POI by determining inflammatory mediator levels, the important mechanism in PPOI pathophysiology, remain limited. Zhu et al. found that peritoneal levels of IL-1, IL-6, and procalcitonin were higher in PPOI patients [10], indicating localized parameters are sensitive for PPOI prediction. Clinical data also found that IL-6 levels are higher in patients undergoing open surgery when compared with patients undergoing laparoscopic surgery [21], while open procedures had been demonstrated to delay recovery of POI [8].

In this study, we report a prospective cohort investigating the association between the inflammatory cytokines and the postoperative complications. We found that systematic changes of IL-6 predicted infectious complications but did not predict PPOI after colorectal surgery. In contrast to many previous animal studies, our results indicate that systematic cytokine levels yield poor predictive value in PPOI diagnosis. This can be partly explained by inevitable confounding factors (e.g., sex, age, type of surgery, and preoperative risk factors) in patient subjects which dilute the influence of POI on systematic inflammatory response [25], while those factors are usually controlled in animal studies. Nevertheless, we believe that our included patients properly represent the common colorectal patient population. An ideal parameter should be able to identify the high-risk patients. In addition, many animal models used in POI research have very different inflammatory response compared with human [26, 27]. For instance, different from the animal data, our study found that systematic TNF-*α* and IL-1*β* levels were extremely low, which was also reported by Ellebæk et al. [28]. In addition, our previous meta-analysis also found that in peritoneal samples, IL-6 is already significantly higher in CAL patients on POD 1, while elevation of TNF-*α* and IL-1*β*, both at much lower concentration, was not observed in the first 3 postoperative days [29].

As is shown in our results, cytokine levels are individually dependent. This has not yet been previously investigated in surgical patients. Picotte et al. also reported great variation in systematic IL-6 levels [30]; therefore, we chose ratio instead of absolute levels of cytokines to rule out the individual baseline variations, which resulted in a higher diagnostic value of the infectious complications than CRP in the ROC analysis.

Based on our results, it seems that only in severe complications but not PPOI can the overwhelming inflammatory response be detected in serum in clinical settings. For those complications, leukocyte count and CRP are commonly used to assist an early diagnosis [31, 32]; thus, we also included them into our analysis. In accordance to the previous studies, our data also support the value of CRP in the diagnosis of infectious complications. Nevertheless, the ROC analysis further demonstrates that IL-6 yielded better diagnostic value than CRP in predicting the infectious complications. It is important to note that the diagnostic value of IL-6 became evident very early on POD 1. All patients had a ratio higher than 1.93 developed infectious complications, indicating the importance of IL-6 evaluation as a promising innovative biomarker for clinical practice.

Although many previous studies exclude patients with other complications from the PPOI group, we still included them to represent a common patient population. This is because it is possible to exclude those patients with complications (e.g., anastomotic leakage) from the POI or PPOI group in a retrospective database. But in a prospective cohort or clinical practice, a surgeon has to differentiate POI or PPOI from other severe complications that require more invasive interventions because many infectious complications first manifest abdominal symptoms before systematic manifestations. This may explain the significantly higher rate of the complications in the PPOI patients in our data.

## 5. Conclusion

POI remains the most common complication after gastrointestinal surgery, without a satisfactory parameter for its early detection or prediction. In this study, we report a prospective cohort study investigating the association between inflammatory cytokines and postoperative complications. We found that serum IL-6 changes predict the infectious complications but not PPOI after colorectal surgery. How to translate knowledge from rodent POI studies to clinical practice is evidently an urgent issue to be addressed. Further exploration of IL-6 seems promising and may assist an early detection of the infectious complications after surgery.

## Figures and Tables

**Table 1:** The variables and definitions of complication and outcome.

Complications/outcome	Definition
PPOI^∗^	Resolution of POI is defined as passage of feces with good toleration of solid food for at least 24 hours. PPOI is diagnosed if POI is not resolved after postoperative day 5; recurrent POI occurring after an apparent resolution of POI was also defined as PPOI [17, 19].
Anastomotic leakage	Defect of the bowel wall integrity at the anastomotic site. A pelvic abscess close to the anastomosis is also considered as anastomotic leakage. The diagnosed leakage were Grade B or C according to classification of Rahbari et al. [18].
Surgical site infection (SSI)	Erythema requiring initiation of antibiotic treatment or a wound requiring partial or complete opening for drainage of a purulent collection.
Pneumonia	Presentation of clinical symptoms including cough, fever, and dyspnoea or consolidation on chest radiography requiring antibiotic treatment with or without a positive sputum culture.
Urinary tract infection	Presents of clinical symptoms, for example, fever, polyuria, and stranguria requiring antibiotic treatment.
Fascia defect	Dehiscence of the abdominal wall with or without the need for reoperation.
Reoperation	During hospital stay, within 30 days postoperative, or during readmission within 30 days after initial discharge.
Length of hospital stay	Day of admission till the day a patient is ready for discharge; this means patient tolerate solid food and had passage of feces, and pain is adequately in control with oral analgesics.
Readmission	Admission within 30 days after discharge for more than 24 hours.
Mortality	Death occurring during hospital stay or within 30 days postoperative.

^∗^Prolonged postoperative ileus.

**Table 2:** Baseline and surgical characteristic comparison between the PPOI and non-PPOI patients.

	Non-PPOI (%)	PPOI (%)
	*n* = 34	*n* = 13
*Patient characteristics*		
Age (yrs.)	67.6 ± 10.4	71.2 ± 11.2
Gender		
Male	21 (62)	6 (46)
Female	13 (39)	7 (54)
BMI (kg/m^2^)	27.2 ± 4.7	24.7 ± 4.2
ASA score		
I	6 (18)	4 (31)
II	14 (41)	4 (31)
III	9 (26)	1 (8)
IV	0	0
Missing	5 (15)	4 (31)
Cardiac comorbidity	11 (32)	3 (23)
Diabetes mellitus	6 (18)	1 (8)
Smoker	6 (18)	1 (8)
COPD	7 (21)	1 (8)
Use of statins	12 (36)	3 (23)
Use of antihypertensive	12 (36)	7 (54)
Neoadjuvant radiotherapy	2 (6)	0
Chemoradiation	4 (12)	1 (8)
Abdominal surgery in history	12 (35)	3 (23)
*Operation characteristics*		
Type of operation		
Low anterior resection	10 (29)	2 (15)
Sigmoid resection	6 (18)	2 (15)
Right hemicolectomy	9 (26)	8 (62)
Left hemicolectomy	5 (15)	0
Colon transversum resection	1 (3)	1 (8)
Abdominal perineal resection	3 (9)	0
Approach		
Laparotomy	13 (38)	5 (38)
Laparoscopy	20 (59)	7 (54)
Conversion	1 (3)	1 (8)
Stapled versus hand sutured^#^		
Sutured	19 (58)	9 (69)
Stapled	14 (42)	4 (31)
Anastomotic configuration^∗^		
Side-end	10 (29)	5 (42)
Side-side	14 (41)	7 (58)
End-end	6 (18)	0
Stoma construction	11 (32)	2 (13)
Prophylactic drainage	4 (12)	1 (8)
Nasogastric tube^∗∗^	10 (29)	6 (50)

PPOI = prolonged postoperative ileus; non-PPOI = patients without PPOI. Data are *n* (%), mean (SD). BMI = body mass index; ASA = American Society of Anesthesiologists classification; COPD = chronic obstructive pulmonary disease; ^#^ *n* = 33 in non-PPOI, *n* = 13 in PPOI group; ^∗^ *n* = 30 in non-PPOI group, *n* = 12 in PPOI group; ^∗∗^ *n* = 12 in PPOI group.

## Supplementary Materials

Supplementary Materials Table S1: percentage of positive samples in the non-PPOI and PPOI group. Table S2: absolute level of cytokine in plasma of the positive samples. Table S3: baseline and surgical characteristic comparison between patients with infectious complications and normal recovery.
