# Supplementary material for: Systemic Inflammatory Cytokines Predict the Infectious Complications but Not Prolonged Postoperative Ileus after Colorectal Surgery
Source: Mediators Inflamm. 2018 Mar 6;2018:7141342. doi: 10.1155/2018/7141342 (PMC5859856; doi:10.1155/2018/7141342)
Supplement: Supplementary Materials — Table S1: percentage of positive samples in the non-PPOI and PPOI group. Table S2: absolute level of cytokine in plasma of the positive samples. Table S3: baseline and surgical characteristic comparison between patients with infectious complications and normal recovery. [file 7141342.f1.pdf]

Supplementary data:

Table S1. Percentage of positive samples in the no-PPOI and PPOI group.

	no-PPOI			PPOI		
	pre-OK (n=31)	POD1 (n=34)	POD3 (n=34)	pre-OK (n=13)	POD1 (n=13)	POD3 (n=13)
Positive samples (%)						
IL-6	100	100	100	100	100	100
IL-1β	25.8	29.4	32.4	15.4	7.7	15.4
TNF-α	12.9	20.6	35.3	7.7	30.8	46.2

Table S2. Absolute level of cytokine in plasma of the positive samples

		no-PPOI			PPOI		
		pre-OK (n=31)	POD1 (n=34)	POD3 (n=34)	pre-OK (n=13)	POD1 (n=13)	POD3 (n=13)
IL-6	Median	377	418	498	100	126	219
IL-1β	Median	13	14	13	12	15	14
TNF-α	Median	163	40	30	67	78	103

no significantly differences

Table S3. Baseline and surgical characteristic comparison between patients with infectious complications and normal recovery.

	No infectious complication	Infectious complication
	**n=24**	**n=22**
Patient characteristics		
Age (years)	70.5 ± 10.3	68.8 ± 11.1
Gender		
male	15 (63)	12 (55)
female	9 (37)	10 (45)
BMI (kg/m2)	26.1 ± 5.0	26.9 ± 4.2
ASA score		
I	5 (21)	5 (23)
II	11 (46)	7 (32)
III	5 (21)	5 (23)
IV	0	0
missing	3 (13)	5 (23)
Cardiac comorbidity	10 (42)	4 (18)
Diabetes Mellitus	3 (13)	4 (18)
Smoker	3 (13)	4 (18)
COPD	3 (13)	5 (23)
Use of statins	7 (29)	8 (36)
Use of antihypertensiva	9 (38)	10 (45)
Neoadjuvant radiotherapy	1 (4)	1 (5)
Chemoradiation	3 (13)	2 (9)
Abdominal surgery in history	9 (38)	6 (27)
Operation characteristics		
Type of operation		
low anterior resection	7 (29)	5 (23)
sigmoid resection	2 (8)	5 (23)
hemicolectomy right	9 (38)	8 (36)
hemicolectomy left	5 (21)	0
colon transversum resection	1 (4)	1 (5)
APR	0	3 (14)
Approach		
laparotomy	7 (29)	10 (45)
laparoscopy	16 (67)	11 (50)
conversion	1 (4)	1 (5)
Stapled vs. hand sutured*		
sutured	17 (71)	10 (48)
stapled	7 (29)	11 (52)
Anastomotic configuration**		
side-end	6 (27)	8 (38)

side-side	13 (59)	8 (38)
end-end	3 (14)	3 (14)
Stoma	6 (25)	7 (32)
Profylactive drainage	1 (4)	4 (18)
Nasogastric tube*	8 (33)	8 (36)

Data are n (%), mean (SD). BMI=Body Mass Index, ASA= American Society of Aneshtesiologists classification.

* n=21 in infectious group

** n=22 in no infectious group and n=21 in infectious group
